# Open access intrapartum CTG database

**DOI:** 10.1186/1471-2393-14-16

**Published:** 2014-01-13

**Authors:** Václav Chudáček, Jiří Spilka, Miroslav Burša, Petr Janků, Lukáš Hruban, Michal Huptych, Lenka Lhotská

**Affiliations:** 1 Department of Cybernetics, Faculty of Electrical Engineering, Czech Technical University in Prague, Prague, Czech Republic; 2 Obstetrics and Gynecology clinic, University Hospital, Brno, Czech Republic

**Keywords:** Cardiotocography, Intrapartum, CTG, Database, Signal processing, Fetal heart rate

## Abstract

**Background:**

Cardiotocography (CTG) is a monitoring of fetal heart rate and uterine contractions. Since 1960 it is routinely used by obstetricians to assess fetal well-being. Many attempts to introduce methods of automatic signal processing and evaluation have appeared during the last 20 years, however still no significant progress similar to that in the domain of adult heart rate variability, where open access databases are available (e.g. MIT-BIH), is visible. Based on a thorough review of the relevant publications, presented in this paper, the shortcomings of the current state are obvious. A lack of common ground for clinicians and technicians in the field hinders clinically usable progress. Our open access database of digital intrapartum cardiotocographic recordings aims to change that.

**Description:**

The intrapartum CTG database consists in total of 552 intrapartum recordings, which were acquired between April 2010 and August 2012 at the obstetrics ward of the University Hospital in Brno, Czech Republic. All recordings were stored in electronic form in the OB TraceVue®;system. The recordings were selected from 9164 intrapartum recordings with clinical as well as technical considerations in mind. All recordings are at most 90 minutes long and start a maximum of 90 minutes before delivery. The time relation of CTG to delivery is known as well as the length of the second stage of labor which does not exceed 30 minutes. The majority of recordings (all but 46 cesarean sections) is – on purpose – from vaginal deliveries. All recordings have available biochemical markers as well as some more general clinical features. Full description of the database and reasoning behind selection of the parameters is presented in the paper.

**Conclusion:**

A new open-access CTG database is introduced which should give the research community common ground for comparison of results on reasonably large database. We anticipate that after reading the paper, the reader will understand the context of the field from clinical and technical perspectives which will enable him/her to use the database and also understand its limitations.

## Background

### Introduction

Fetal heart activity is the prominent source of information about fetal well being during delivery. Cardiotocography (CTG) – recording of fetal heart rate (FHR) and uterine contractions – enables obstetricians to detect fetus with deteriorating status (e.g. ongoing fetal hypoxia), which may occur even in a previously uncomplicated pregnancy. Even though fetus has its own natural defense mechanism to tackle the oxygen insufficiency during the delivery, in some cases only timely intervention can prevent adverse consequences. Hypoxia, with prevalence lying in the region of 0.6% [1] to 3.5% [2], is considered to be the third most common cause of newborn death [3].

Cardiotocography was introduced in late 1960s and is still the most prevalent method of intrapartum hypoxia detection. It did not, however, bring the expected improvements in the delivery outcomes in comparison to previously used intermittent auscultation [4]. Moreover, continuous CTG is the main suspect for growing percentage of cesarean sections (CS) for objective reasons, which increased in the last decade [5].

To tackle the problems of CTG three principal approaches were followed during the years. The first approach focused on improving low inter and intra-observer agreement [6]. In 1986 International Federation of Gynecology and Obstetrics (FIGO) introduced general guidelines [7] based on evaluation of macroscopic morphological FHR features and their relation to the tocographic measurement. But even though the guidelines are available for almost thirty years now, poor interpretation of CTG still persists [8-10]. Many tweaks to the guidelines were proposed during the years, but with no significant change to the inter-observer variability. For overview of the different guidelines we refer the reader to [11].

The second approach to improve the overall results of CTG looks for technical solutions to add more objective/quantitative evaluation of the state of the fetus using additional measurement techniques. Some used techniques are regionally/country limited in their use – such as fetal blood sampling [12,13] or fetal pulse oxymetry [14]. The only widespread method is evaluation of averaged repolarization behavior of fetal ECG performed by STAN. Many studies were published comparing STAN-enhanced CTG with use of CTG only. The majority of studies proved that the addition of STAN indeed leads to slightly better fetal outcomes [15,16] but problems were also reported [17,18].

Attempts to computer evaluation of the CTG – the third approach – started almost immediately after introduction of the first unified FIGO guidelines. FIGO features became fundamental in most of the first clinically oriented approaches and automatically extracted morphological features played the major role in all automatic systems for CTG analysis [19,20].

We suggest interested reader to refer to e.g. [21] for more details on CTG preprocessing and to e.g. [22] for more details on different features that had been extracted to characterize the FHR since the focus of this paper is rather on the database presentation.

When reviewing literature on automatic CTG processing, two things are striking. First, there is a large disconnection between approaches and goals in the clinical and technical papers. The clinical papers are mostly looking for applicable solutions to the clinically pressing issues (lack of agreement, critically misclassified recordings). The technical papers often use CTG data as just an another input to the carefully tuned classifiers. Most works use very small ad-hoc acquired datasets, differently sampled with various parameters used as outcome measures, though we have to concede that our previous works [22,23] were done exactly in the same manner. It is hard to believe that it is more than 30 years when computer processing of CTG has begun [24] and since then, no common database of CTG records is available^a^. There is no way how to compare/improve/disregard among different results. And that, in our opinion, hinders any significant progress towards the ultimate goal of a usable and working automated classification of the CTG recordings.

In this paper we present a novel open-access CTG database, which we will call further in the paper CTUUHB database^b^. It consists of CTG records and clinical information. We first provide a comprehensive overview of databases used in literature in the last years. Then we describe development of the CTU-UHB database. We discuss the criteria for selection of records for the database from clinical and technical point of view. At last, we present a detailed description of main clinical and technical parameters, which, in our opinion, are important for understanding and should be taken into account when using the database.

### Overview of CTG databases used in literature

We performed a systematic search in the electronic database PUBMED including records up to February 2013. The language of studies was restricted to English. The various combination of the following keywords were used: cardiotocography, fetal heart rate, fetal heart rate monitoring, computer assisted diagnosis, fetal distress, asphyxia, hypoxemia, fetal hypoxia. In the selected articles the references were searched and articles that cited the paper were searched as well.

It is impossible to provide exhaustive review and, therefore, several inclusion criteria were applied to include all relevant works but keep the overview as clear as possible. First, if a CTG database was used in multiple works, we included the paper where the database was described in most detail. If the description was the same, we included the most recent paper, e.g. we preferred paper of Jezewski et al. [25] rather than of Czabanski et al. [26]. Second, only those works that used intrapartum CTG signals were considered, e.g. we did not include the work of H. Ocak [27] since he worked with UCI Cardiotocography Data Set^c^. Third, we preferred journal papers and works that attempted to show results with regards to objective annotation (pH, base excess, etc.).

Our search of CTG databases used in other studies (with applied selection criteria) resulted in inclusion of 22 works. Due to the space limitation the overview had to be split into two tables, Tables 1 and 2. Table 1 presents used databases regarding the CTG signals and clinical parameters, namely: type of acquisition (ultrasound Doppler (US), direct fetal electrocardiogram measurement (FECG)); antepartum (ante.) or intrapartum (inte.) phase; stage of labor (I. or II.); length of FHR signal; time to actual delivery; use of uterine contractions (UC), description of inclusion criteria; description of clinical data; evaluation type: objective (obj.), subjective (subj.), or combination of both (comb.); number of total cases. The number of cases varies from study to study, the lowest being around 50 cases, and the highest being 7568 cases. Table 2 presents the overview of databases from classification point of view. It is apparent that in each paper different criteria for classes division were used, thus, making any comparison of results between different studies virtually impossible.

**Table 1:** Overview of databases used in various works I

**Reference**	**Acquisition**	**Timing**	**Labor stage**	**FHR sig. used**	**Time to delivery**	**UC used**	**Incl.**	**Clinical**	**Evaluation**	**# Total**
				**[min]**	**[min]**		**criteria**	**info.**	**type**	**cases**
Nielsen et al. 1988 [28]	N/A	intra.	I.	30	N/A	Yes	No	No	obj.	50
Chung et al. 1995 [29]	FECG	intra.	N/A	N/A	N/A	Yes	Yes	Yes	obj.	73
Keith et al. 1995 [30]	N/A	intra.	N/A	> 120	Until del.	Yes	No	Yes	comb.	50
Bernardes et al. 1998 [31]	US, FECG	ante., intra.	I.,II.	–	Until del.	Yes	No	Yes	obj.	85
Maeda et al. 1998 [32]	N/A	intra.	N/A	50	N/A	No	No	No	subj.	49
Lee et al. 1999 [33]	FECG	intra.	N/A	–	N/A	Yes	No	No	subj.	53
Chung et al. 2001 [34]	US	ante., intra.	I.,II.	N/A	120	No	No	Yes	comb.	76
Strachan et al. 2001 [35]	FECG	intra.	I.,II.	> 30	Until del.	Yes	No	Yes	obj.	679
Siira et al. 2005 [36]	FECG	intra.	I.,II.	60	95% bellow 9	Yes	Yes	Yes	obj.	334
Cao et al. 2006 [37]	US, FECG	intra.	N/A	30	N/A	Yes	No	No	subj.	148
Salamalekis et al. 2006 [38]	US	intra.	I.,II.	N/A	Until del.	No	Yes	Yes	comb.	74
Georgoulas et al. 2006 [39]	FECG	intra.	I.,II.	20–60	Until del.	No	No	No	obj.	80
Gonçalves et al. 2006 [40]	US, FECG	intra.	I.,II.	32–60	Until del.	No	Yes	Yes	obj.	68
Costa et al. 2009 [41]	FECG	intra.	I.,II.	–	Until del.	Yes	Yes	Yes	obj.	148
Elliott et al. 2010 [42]	N/A	intra.	I.,II.	> 180	Until del.	Yes	Yes	Yes	subj.	2192
Warrick et al. 2010 [43]	US, FECG	intra.	I.,II.	> 180	Until del.	Yes	Yes	No	obj.	213
Jezewski et al. 2010 [25]	US	ante., intra.	N/A	–	N/A	Yes	Yes	Yes	obj.	749
Helgason et al. 2011 [44]	FECG	intra.	I.,II.	> 30	Until del.	Yes	No	No	comb.	47
Chudacek et al. 2011 [22]	US, FECG	intra.	I.,II.	20	Until del.	No	Yes	Yes	comb.	552
Spilka et al. 2012 [23]	US, FECG	intra.	I.,II.	20	Until del.	No	Yes	No	obj.	217
Georgieva et al. 2013 [45]	N/A	intra.	I.,II.	–	Until del.	No	Yes	Yes	obj.	7568
Czabanski et al. 2013 [46]	N/A	ante., intra.	N/A	60	N/A	No	Yes	Yes	subj.	2124 ^*‡*^

Legend: “N/A” – information not available, “–” – authors used the whole available FHR signal without specifying the length, ^*‡*^ – 2124 recordings, 333 woman.

**Table 2:** Overview of databases used in various works II

**Reference**	**Classes (categories)**	**Division criteria for classes**	**# classes**	**# cases in classes**	**# total cases**
Nielsen et al. 1988 [28]	Normal; pathological	Apgar 1 min. < 7 or pH < 7.15 or BE < -10	2	34; 16	50
Chung et al. 1995 [29]	Normal; abnormal	pH < 7.15	2	65; 8	73
Keith et al. 1995 [30]	5-tier scoring system	17 clinicians, pH, BDecf, Apgar	5	38; 12	50
Bernardes et al. 1998 [31]	norm.; susp.; pathol.	pH, Apgar, neonatology	3	56; 22; 7	85
Maeda et al. 1998 [32]	norm.; susp.; pathol.	manual clinical rules	3	12; 18; 19	49
Lee et al. 1999 [33]	Normal CTG; decels.	1 clinician	2	N/A	53
Chung et al. 2001 [34]	Normal; presumed distress; acidemic	norm. FHR; abnorm. & pH > 7.15; abnorm. & pH < 7.15	3	36; 26; 14	76
Strachan et al. 2001 [35]	Normal; abnormal	pH ≤ 7.15 & BDecf > 8	2	608; 71	679
Siira et al. 2005 [36]	Normal; acidemic	pH < 7.05	2	319; 15	334
Cao et al. 2006 [37]	reassuring; NR	2 clinicians	2	102; 44	148
Salamalekis et al. 2006 [38]	Normal; NR [NR & pH > 7.20; NR & pH < 7.20]	FIGO, pH < 7.20	2	32; 42	74
Georgoulas et al. 2006 [39]	Normal; at risk	pH > 7.20; pH < 7.10	2	20; 60	80
Costa et al. 2009 [41]	Omniview-SisPorto 3.5 alerts	pH < 7.05	2	7; 141	148
Elliott et al. 2010 [42]	Normal; abnormal	BDecf ≥ 12 & NE	2	60; 2132	2192
Warrick et al. 2010 [43]	Normal; pathological	BDecf < 8; BDecf ≥ 12	2	187; 26	213
Jezewski et al. 2010 [25]	Normal; abnormal	Apgar N/A min. < 7 or birth weight < 10 ^th^ perc. or pH < 7.2	2	28% abnorm.	749
Helgason et al. 2011 [44]	FIGO-TN; FIGO-FP; FIGO-TP	norm. FHR & pH ≥ 7.30; abnorm. & pH ≥ 7.30; abnorm. & pH ≤ 7.05	3	15; 17; 15	47
Chudacek et al. 2011 [22]	norm.; susp.; pathol.	3 clinicians	3	139; 306; 107	552
Spilka et al. 2012 [23]	Normal; pathological	pH < 7.15	2	123; 94	217
Georgieva et al. 2013 [45]	Normal; adverse	pH < 7.1 & neonatology	2	N/A	7568
Czabanski et al. 2013 [46]	Normal; abnormal	Apgar 10 min. < 5	2	306; 27	2124 ^*‡*^

Abbreviations: NR – non-reassuring, NE – neonatal encephalopathy, ^*‡*^ – 2124 recordings, 333 woman.

## Construction and content

### Ethics statement

The CTG recordings and clinical data were matched by anonymized unique identifier generated by the hospital information system. The timings of CTG records were matched to stages of labor (first and second stage) and were made relative to the time of birth, thus also de-identified. This study was approved by the Institutional Review Board of University Hospital Brno; all women signed informed consent.

### Data collection

The data were collected between 27^*th*^ of April 2010 and 6^*th*^ of August 2012 at the obstetrics ward of the University Hospital in Brno (UHB), Czech Republic. The data consisted of two main components, the first were intrapartum CTG recordings and the second were clinical data.

The CTGs were recorded using STAN S21 and S31 (Neoventa Medical, Mölndal, Sweden) and Avalon FM40 and FM50 (Philips Healthcare, Andover, MA) fetal monitors. All CTG signals were stored in an electronic form in the OB TraceVue®;system (Philips) in a proprietary format and converted into text format using proprietary software provided by Philips. Each CTG record contains time information and signal of fetal heart rate and uterine contractions both sampled at 4 Hz. When a signal was recorded using internal scalp electrode it also contained T/QRS ratio and information about biphasic T-wave. From 9164 intrapartum recordings the final database of 552 carefully selected CTGs was created keeping in consideration clinical as well as technical point of view; the details about recordings selection are provided further.

The clinical data were stored in the hospital information system (AMIS) in the relational database. Complete clinical information regarding delivery and fetal/maternal information were obtained. The clinical data included: delivery descriptors (presentation of fetus, type of delivery and length of the first and second stage), neonatal outcome (seizures, intubation, etc.), fetal and neonatal descriptors (sex, gestational week, weight, etc.), and information about mother and possible risk factors. For the final CTU-UHB database, clinical data were exported from relational database and converted into Physionet text format.

### Data selection and criteria considered

**Figure 1:**
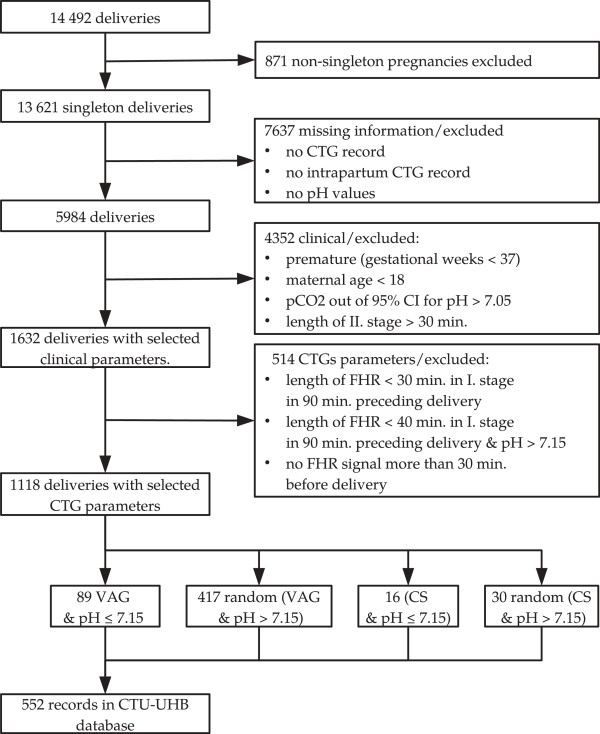
**Selection of recordings for the final database.** Flowchart diagram describing the process of data selection for the final database.

The selection procedure of the records was based on both clinical and CTG signal parameters and the process is shown in Figure 1.

#### Clinical criteria

In the following paragraphs we describe criteria that were used for records exclusion. Additionally we discuss shortly the criteria that were included in the description of the final database but were not used for records exclusion.

##### Clinical selection criteria

The following parameters were taken into account for inclusion of recordings into the final database. References in this section refer to a description of particular parameter. 

• Women’s Age – although the women’s high age plays significant role in the probability of congenital diseases, for the intrapartum period no significance was found [47]. Low age (maternal age <18 years) could have an adverse effect [48] and such records were therefore excluded.

• Week of gestation – maturity of the fetus plays significant role in the shape and behavior of the FHR antepartum as well as intrapartum [49]. Therefore the selection was limited to mature fetuses (weeks of gestation ≥37) according to the last menses counting, which was in majority cases confirmed by ultrasound measurement during antepartum check-ups.

• Known fetal diseases – fetuses with known congenital defects or known intrauterine growth restriction (IUGR) that could influence the FHR and/or outcome of the delivery were excluded from the database. Additionally, postnatally detected defects were consulted and two cases with transposed large veins were left in the set, since these two particular changes should not have influenced the FHR.

• Type of gravidity – only singleton, uncomplicated pregnancies were included.

• Type of delivery – the majority of the database consists of vaginal deliveries. Nevertheless to increase the number of cases with pathological outcome in the database, 16 CS recordings with pH≤7.05 were included and consequently a control group consisting of 30 CS with normal outcomes was also included to enable separate evaluation if necessary.

##### Additional clinical criteria provided

Together with criteria used for selection, the following criteria were considered and are available together with the CTG data: 

• Sex of the fetus – both sexes were included even though the sex of fetus significantly influences the outcome according to Bernardes et al. [50].

• Parity – although the first delivery can be “more difficult” in general clinical sense [51] it is the same from the point of view of the FHR recording.

• Risk factors – to be able to describe and identify the a priori high-risk pregnancies. We have included risk factors that could have influenced the state of the baby before entering the labor. For full review of the parameters and further references we recommend paper of Badawi et al. [52]. The final risk factors included in the database were gestational diabetes, preeclampsia, maternal fever (>37.5°C), hypertension and meconium stained fluid.

• Drugs – especially those administered during delivery were considered only with regard to their influence on FHR. Opiates may influence the FHR directly but are rarely used in the Czech Republic during delivery and were not used in any of the cases included in the database. Therefore, we do not provide information about drugs administration in the database. Note that e.g. oxytocin used for enhancement of the uterine activity influences the FHR in majority indirectly, via increase of uterine activity, and thus can be assessed from the CTG alone.

• Other criteria – complementary information in order to offer insight why e.g. operative delivery was chosen. These include: induced delivery, type of presentation (occipital/breech), no progress of labor, dystocia cephalocorporal (incoordinate uterine activity), dystocia cephalopelvic.

#### Labor outcome measures

Since our main intention was to prepare database that could be used for comparison of different automated approaches we have selected only those recordings that included umbilical artery pH. We added all additional outcome measures that were available for the recording in the hospital information system. Some of these measures are often misused and we will discuss their disadvantages below.

##### Outcome measure selection criteria

To enable objective classification the pH measure was considered as essential for the evaluation of the database. 

• Umbilical artery pH (pH) – is the most commonly used outcome measure, sign of respiratory hypoxia. Records with missing pH were excluded. Following suggestion by Rosen [53] records, which had values of *pCO*_2_ outside 95^*th*^ percentile [54] were excluded except those with pH≤7.05, which even according to [54] should be approached with care.

##### Additional outcome measures provided

Even though the is pH is the most commonly used measure, we included additional measures such as following: 

• Base excess (BE) – is often used in the clinical setting as a sign for metabolic hypoxia, but is often false positive [53].

• Base deficit in extracellular fluid (BDecf) – is according to Rosén et al. [53] a better measure of metabolic hypoxia than BE. Still pH remains more robust measure and according to last study of Georgieva et al. remains the most informative [45].

• Neonatology – complete neonatological reports were acquired for all the cases in pre-prepared database. No severe cases of neonatal morbidity were found, no hypoxic ischemic encephalopathy, no seizures (for details on neonatal morbidity see [55]).

• Subjective evaluation of the outcome of the delivery based on Apgar’s score (Apgar), where five categories are used to assess the newborn child in 1^*s* *t*^, 5^*th*^ and 10^*th*^ minute [56].

The complete database was used for inter-intra observer variability study. In this study 9 senior obstetricians evaluated CTG signals. The clinical evaluation will be added to the database as soon as processed.

#### Signal criteria

When the data were filtered according to the clinical information, we have applied the following criteria on CTG records: 

• Signal length – we have decided to include 90 minutes preceding the delivery, where the delivery time represents also the time when the objective (pH, etc.) evaluation of labor was acquired. 

I. stage – the length of the I. stage was limited to a maximum of 60 minutes in order to keep recordings easily comparable. The minimal length was dependent on the pH of the records in question – to include as much abnormal records as possible. Thus the minimal length of the I. stage of 30 minutes was required for recording with pH ≤7.15 and 40 minutes for others. The time distance from the end of the I.stage to birth was not allowed to be larger than 30 minutes.

II. stage – based on our previous experience with analysis of the II. stage of labor (active pushing phase), we limited the II. stage to 30 minutes at maximum. This also limits the possibility of adverse events occurring in the II. stage, which could disconnect CTG recording in the I. stage with objective evaluation of the delivery.

• Given the restriction above the signals are 30(40)–90 minutes long depending on a) the length of the II. stage and also b) available signal in the I. stage. No signal ends earlier than 30 minutes before delivery.

• Missing signal – amount of missing signal was, except for the II. stage, kept to possible minimum. Nevertheless the trade-off between having full signal and having recordings with abnormal outcomes had to be made. No more than 50 % of signal was allowed to be missing in the I. stage.

• Noise and artifacts – these are a problem especially for the recordings acquired by the ultrasound probe. Certainly in some recordings maternal heart rate is intermittently present. But even though it can pose a challenge for user of the database it also reflects the clinical reality.

• Type of measurement device – the database is composed as a mixture of recordings acquired by ultrasound doppler probe, direct scalp measurement or combination of both – again reflecting the clinical reality at the obstetrics ward of UHB.

### Description of the Database

**Figure 2:**
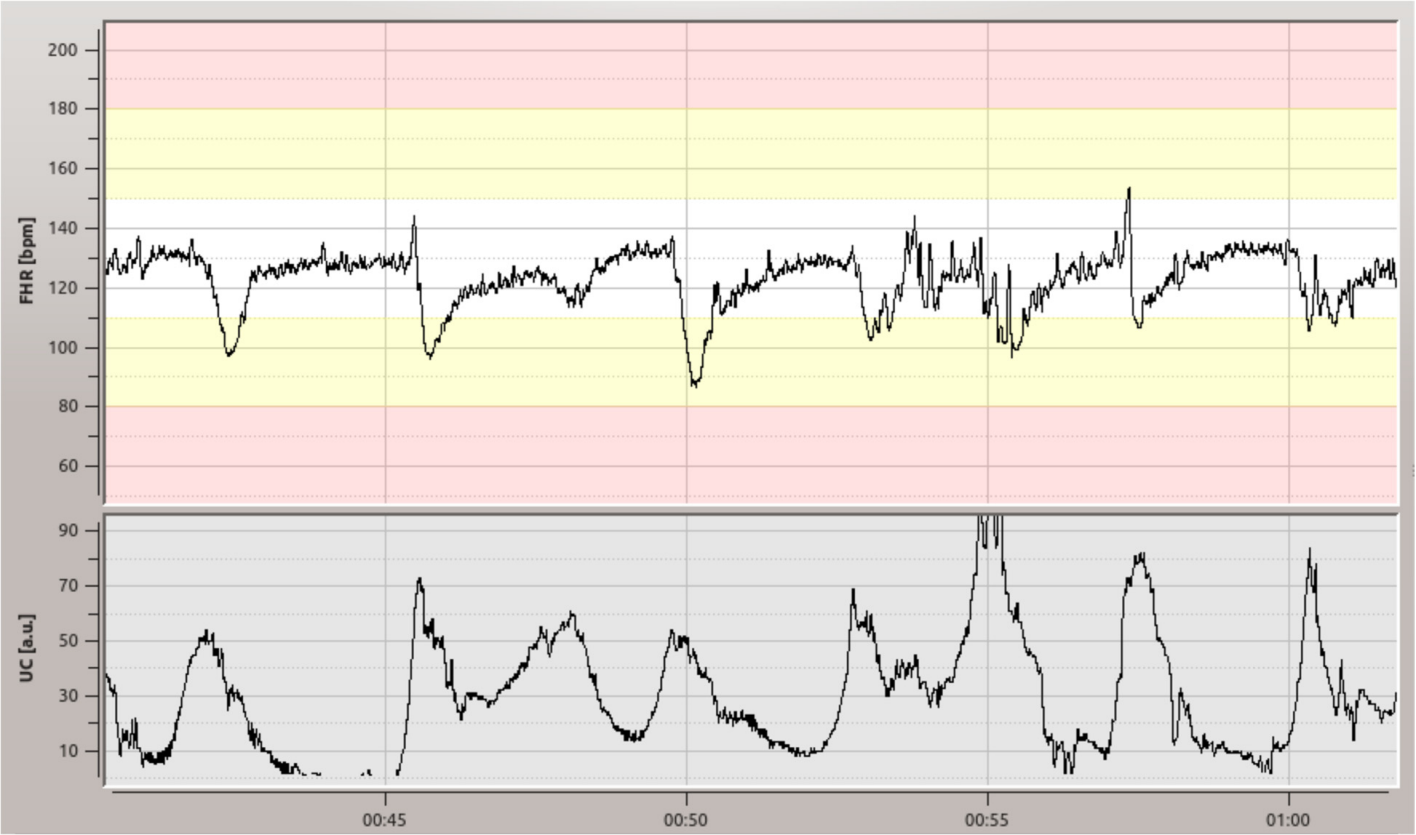
**Record of fetal heart rate (FHR) and uterine contractions (UC).** An example record from the database. Fetal Heart Rate at the top, and uterine contractions at the bottom. The end of I. stage of labor is marked with blue line and an arrow.

Records for the CTU-UHB database were selected based on clinical and technical criteria described above. Table 3 provides overview of patient and labor outcome measure statistics and Table 4 presents main parameters regarding the CTG signals. The CTG signals were transformed from proprietary Philips format to open Physionet format [57], all data were anonymized at the hospital and de-identified (relative time) at the CTU side. An example of one CTG record is shown in Figure 2.

**Table 3:** Patient and labor outcome statistics for the CTG-UHB cardiotocography database

**506 – Vaginal (44 – operative); 46 – Caesarean Section**				
**US = 412; DECG = 102; US-DECG = 35; N/A = 3**				
	**Mean (Median)**	**Min**	**Max**	**Comment**
Maternal age (years)	29.8	18	46	Over 36y: 40.
Parity	0.43 (0)	0	7	
Gravidity	1.43 (1)	1	11	
Gestational age (weeks)	40	37	43	Over 42 weeks: 2
pH	7.23	6.85	7.47	
BE	-6.36	-26.8	-0.2	
BDecf (mmol/l)	4.60	-3.40		
Apgar 1min	8.26 (8)	1	10	AS1 < 3: 18
Apgar 5min	9.06 (10)	4	10	AS5 < 7: 50
Neonate’s weight (g)	3408	1970	4750	SGA: 17; LGA: 44
Neonate’s sex (F/M)	259 / 293			

Abbreviations: AS1, AS5 – Apgar score at 1^*st*^ and 5^*th*^ minute respectively; SGA, LGA – fetus small, large for gestational age.

**Table 4:** CTG signal statistics for the CTG-UHB cardiotocography database

**506 – Vaginal (44 – operative); 46 – Caesarean Section**				
**US = 412; DECG = 102; US-DECG = 35; N/A = 3**				
	**Mean**	**Min**	**Max**	**Comment**
Length of I. stage (min)	225	45	648	
Length of II. stage (min)	11.87	0	30	
Dist. SignalEnd to Birth (min)	2.70	0	29	Over 10 min: 9
Noisy data W1 (%)	12.38	0	74	
Missing data W1 (%)	3.59	0	87	
Overall W1 (%)	15.98	0	89	Over 50%: 18
Noisy data W2 (%)	13.42	0	49	
Missing data W2 (%)	0	0	0	
Overall W2 (%)	13.14	0	49	Over 25%: 98
Noisy data II. stage (%)	22.62	0	91	
Missing data II. stage (%)	8.47	0	100	
Overall II. stage (%)	31.26	0	100	Over 50%: 97

W1 – 30 minute-long window beginning 60 minutes before end of the first stage of labor, W2 – 30 minute-long window beginning 30 minutes before end of the first stage of labor.

#### CTG database – vaginal deliveries

The main part of the CTG database consists of 506 intrapartum recordings delivered vaginally. It means the deliveries got always to the II. stage of labor (fully dilated cervix, periodical contractions), even though not all deliveries had active pushing period. Some were delivered operatively by means of forceps or vacuum extraction (VEX). The main outcome measures are presented in Tables 3 and 4.

Please note the column “Comment”, which gives additional information either with regard to the number of potential outliers or points out interesting features of the database such as number of pathological cases based on certain parameters or quality of the recording in each window.

#### CTG database – deliveries by Caesarean Section

The database was selected to have the majority of intrapartum recordings with vaginal delivery. Nevertheless due to low number of cases with severely-abnormal outcomes, we have decided to add all recordings delivered by Cesarean Section (CS) with abnormal outcomes that conformed with the requirements mentioned above (16 CS records). Additional 30 CS recordings with normal outcome were randomly selected and added as a control-group. This control should enable the user of the database to evaluate CS recordings separately, if necessary. The details of the CS part of the database can be found in Tables 3 and 4.

#### Additional perspectives on the database

In addition to the tables presented above four more tables are included in the Additional files section. Additional file 1: Table S5 and Additional file 2: Table S6 show the structure of the database with respect to umbilical cord artery pH values. The main reason for presentation of theses tables is to allow clear visibility of the features’ values and their change with different pH. We are well aware of the article of Yeh [58], which states that there is weak relation between umbilical artery pH after delivery and negative outcome to the baby. Nevertheless the pH still remains the main “objective” parameter – a summary of the delivery – as clearly presented in [45]. Finally, two short Tables S7 and S8 in the Additional file 3 describe different risk factors presented in the database again related to umbilical cord artery pH on vaginal and CS part of the database, respectively.

## Utility and discussion

The CTU-UHB database is the first open-access database for research on intrapartum CTG signal processing and analysis. In the following paragraphs we will highlight the subjects, that could, if unobserved, lead to problems with use of the database.

The CTU-UHB users should be aware that there is a possible noise in the clinical data, since some information had to be mined from free text. Even though the whole data was carefully checked it is possible that some noise is present. However, this noise should not significantly disrupt any results obtained. Also we note that, due to the selection process, the database is biased from normal population (disproportional amount of low pH deliveries etc.) but this bias is evident in all other studies and, more importantly, if we would keep the database in the original form, the potential users would be forced to select the data themselves – resulting in different selection criteria and making, again, any comparison across studies infeasible.

From Table 2 it is evident that each study used different outcome measures, or their combinations. Again, this makes any comparison across studies infeasible. There are two main sources of evaluation: objective by e.g. umbilical artery pH, which is prominent example, and subjective evaluation by experts according to their knowledge and/or guidelines used. For the clarity reasons we will focus on non-expert outcome measures, as those are discussed in another recently prepared paper of our research group (Spilka et al.: Analysis of CTG interpretation by expert-obstetricians, submitted).

Exact relation of umbilical pH after delivery to CTG/FHR is so far not fully understood, time between the recording and actual delivery plays a crucial role, and it seems that pH is only weakly correlated to clinical annotation [59,60]. The best example is the timely CS due to suspect CTG – the CTG is suspect/pathological but the intervention prevented the baby to get into real asphyxia that would be reflected in the pH value. Yeh [58] claims that there is only weak relation between pH and actual outcome for the well-being of the baby. Following work [45] on the same database relates low values of pH to higher risk of clinical complications. From the studies on cerebral palsy in neonates pH and BDecf are recommended as preferred measures [61] even though [62] says the opposite. Additionally intrapartum events and cerebral palsy are very rarely related by the intrapartum hypoxia only [63] and the real outcome of the delivery can be seen only in several years-long follow up [64].

There is no general agreement on the threshold, which should be used to distinguish between the normal and abnormal outcome of the delivery. There are several used/supported possibilities. 

• Cerebral Palsy – pH ≤7 together with BDecf ≤12 [65] was found to be related to significant increase of the cerebral palsy.

• Pathological – pH ≤7.05 [16] is used as a threshold by most studies. Even though this value is not used unanimously, it is generally accepted as the threshold between pathological and not-pathological delivery outcomes. Combination with BDecf was used e.g. in [66].

• Abnormal (lower than normal) pH <7.10 [67,68] – this value is supported by recent works on the large Oxford database as well as used heuristically at the UHB as a sign of severe problems with the delivery.

• Abnormal (lower than normal) pH <7.15 [69,70].

• Abnormal (lower than normal) pH <7.20 [71] this particular value is also mentioned as an threshold to abnormal outcome pH by Irelands’ obstetrician guidelines.

Regarding the Base deficit/base excess values the BDefc established by [72] is the only usable measure for assessment of metabolic hypoxia [53]. Nevertheless in many papers as well as in the clinical practice the base excess (BE) is used erroneously [53].

In general pH is more robust but is affected more by respiratory asphyxia, BDecf is more about metabolic asphyxia. Regarding the reliability of the objective measurements they are in general much more reliable than any expert opinion. Nevertheless biochemical measures are very dependent on the measuring procedure – pH is in general considered to be more robust than the BDecf where it is necessary to use consideration about the *pCO*_2_ measurements [54].

Among undocumented parameters in the database, which could influence the shape and/or different properties of FHR one could count e.g. smoking [73], which can increase the heart rate, or epidural analgesia [74,75] responsible for intermittent fetal bradycardia due maternal intermittent hypotension. Some risk factors can influence the look of the FHR such as diabetes mellitus, where FHR looks more immature [76]. Also technical parameters can influence the FHR itself – such as size of autocorrelation window for deriving FHR from ultrasound [77], or the derived parameters – such as power spectral density (PSD) of FHR, which can be affected by the type of interpolation [78].

Length of the data used is usually limited by the availability of the data. Really long signals (spanning from the check-in to delivery) enable us to create individualized approach to each fetus with regard to its starting point [53]. We have much more information to analyze, which can be positive [79] or confusing based on the point of view [80]. Short signals such as e.g. 70 min long [81] enables us to try to find direct relation between the features measured and the outcome.

Another question is how to treat the II. stage of labor. Will the length of it confuse the extracted features? General opinion on the second stage is that it is different from the I. stage – in shape of the signal. It is also very often noisy and it differs even in the clinical treatment where obstetricians are much more lenient to apply operative delivery in case of unclear trace [82].

## Conclusion

The CTU-UHB database is the first open-access database available for research on intrapartum CTG signal processing and analysis. It is available at the Physionet website. The database is reasonably large and allows researchers to develop algorithms/methods for CTG analysis and classification. Using CTU-UHB database – different approaches can be easily compared one with another in the objective fashion. Intuitively, the use of common database can stimulate the research in CTG signal processing and classification and move the community to the ultimate goal – an automatic analysis of CTG and its possible extension to a decision support system for clinicians.

## Availability and requirements

The database is published at http://physionet.org/physiobank/database/ctu-uhb-ctgdb/ as an open-access database on the website dedicated to research in cardiology, heart rate variability and related fields.

The database is free to use for non-commercial purposes given that any publication using the database refers to this paper.

## Endnotes

^a^ The only published attempt in this direction was found in [83], but it was discontinued since.

^b^ Czech Technical University – University Hospital Brno.

^c^ UCI Cardiotocography Data Set includes only CTG features not the signals – for more details see [84].

## Abbreviations

CTG: Cardiotocography; FHR: Fetal heart rate; FECG: Fetal electrocardiography; UC: Uterine contractions; US: Ultrasound; BE: Base excess; BDecf: Base deficit in extracellular fluid; PSD: Power spectral density.

## Competing interests

The authors declare that they have no competing interests.

## Authors’ contributions

All authors of the manuscript made a substantial contributions to the design of the study, data collection and analysis, results interpretation, to the article revising and final approval, namely: VC, JS, PJ and LH have formed the main outline of the study and specified the methodology and designed the database. VC, JS, MB and MH have been involved in the acquisition, cleaning and preprocessing of the data. PJ and LH have reviewed and finally selected clinical features to be used for description of the data. LL helped with methodology and paper organization. VC, JS, LL and PJ drafted the manuscript. All authors read and approved the final manuscript.

## Pre-publication history

The pre-publication history for this paper can be accessed here:

http://www.biomedcentral.com/1471-2393/14/16/prepub

## Supplementary Material

Additional file 1 **Table S5.** Main clinical parameters of the vaginal delivery part of the CTG database and its relation to pH. Click here for file

Additional file 2 **Table S6.** Main clinical parameters of the CS part of the CTG database and its relation to pH. Click here for file

Additional file 3 **Table S7 and Table S8.** Clinical parameters (risk factors and means of measurement) - vaginal delivery part of the CTG database - pH related. Presentation: O stands for occipital and B for breech.
**Table S8** Clinical parameters (risk factors and means of measurement) - sectio caesarea delivery part of the CTG database - pH related. Presentation: O stands for occipital and B for breech. Click here for file
