# Supplementary material for: Open access intrapartum CTG database
Source: BMC Pregnancy Childbirth. 2014 Jan 13;14:16. doi: 10.1186/1471-2393-14-16 (PMC3898997; doi:10.1186/1471-2393-14-16)
Supplement: Additional file 1 — Table S5. Main clinical parameters of the vaginal delivery part of the CTG database and its relation to pH. [file 1471-2393-14-16-S1.PDF]

Additional files – Table 5: Main clinical parameters of the vaginal delivery part of the CTG database – pH related.

	**pH ≤ 7.05**<br>**# cases: 38**			**pH > 7.05**<br>**# cases: 468**			**pH > 7.15**<br>**# cases: 412**			**pH > 7.25**<br>**# cases: 261**		
	**Mean**	**Min**	**Max**	**Mean**	**Min**	**Max**	**Mean**	**Min**	**Max**	**Mean**	**Min**	**Max**
Maternal age (years)	29.97	19	42	29.79	18	46	29.76	18	43	30.08	18	43
Parity	0.18	0	2	0.45	0	7	0.48	0	7	0.56	0	5
Gravidity	1.37	1	5	1.44	1	11	1.47	1	11	1.57	1	11
Gestational age (weeks)	39.97	38	42	40.00	37	43	39.98	37	43	40.03	37	43
pH	6.98	6.85	7.04	7.25	7.05	7.47	7.27	7.15	7.47	7.31	7.25	7.47
BE	-16.63	-26.80	-8.30	-5.68	-13.30	-0.20	-5.07	-11.70	-0.20	-4.12	-8.10	-0.20
BDecf (mmol/l)	13.74	6.76	26.11	3.99	-3.40	10.92	3.51	-3.40	9.70	2.81	-3.41	7.03
Apgar 1min	6.53	1	10	8.4	1	10	8.53	2	10	8.84	3	10
Apgar 5min	7.92	4	10	9.15	4	10	9.25	4	10	9.43	4	10
Neonate's weight (g)	3357	2570	4200	3412	1970	4750	3411	1970	4750	3404.6	1970	4750
Neonate's sex (F/M)		16 / 22			223 / 245			195 / 217			124 /137	
# cases with pat. outcome	BE: 25; BDecf: 18<br>Apgar at 1min: 6<br>Apgar at 5min: 11			BE: 4; BDecf: 0<br>Apgar at 1min: 11<br>Apgar at 5min: 33			BE: 0; BDecf: 0<br>Apgar at 1min: 9<br>Apgar at 5min: 24			BE: 0; BDecf: 0<br>Apgar at 1min: 2<br>Apgar at 5min: 7		
