# Supplementary material for: Open access intrapartum CTG database
Source: BMC Pregnancy Childbirth. 2014 Jan 13;14:16. doi: 10.1186/1471-2393-14-16 (PMC3898997; doi:10.1186/1471-2393-14-16)
Supplement: Additional file 2 — Table S6. Main clinical parameters of the CS part of the CTG database and its relation to pH. [file 1471-2393-14-16-S2.PDF]

Additional files – Table 6: Main clinical parameters of the SC delivery part of the CTG database – pH related.

	**pH $\leq$ 7.05 # cases: 9**			**pH $>$ 7.05 # cases: 37**			**pH $>$ 7.15 # cases: 32**			**pH $>$ 7.25 # cases: 22**		
	**Mean**	**Min**	**Max**	**Mean**	**Min**	**Max**	**Mean**	**Min**	**Max**	**Mean**	**Min**	**Max**
Maternal age (years)	27.44	21	34	29.7	20	46	29.25	20	39	30.32	20	39
Parity	0	0	0	0.35	0	2	0.32	0	2	0.55	0	2
Gravidity	1	1	1	1.62	1	8	1.72	1	8	2.05	1	8
Gestational age (weeks)	40.33	38	41	39.8	37	43	39.84	37	43	39.86	37	43
pH	6.99	6.95	7.04	7.26	7.08	7.43	7.28	7.15	7.43	7.32	7.25	7.43
BE	-18.67	-23.70	-12.00	-6.17	-12.10	-1.30	-5.32	-10.40	-1.30	-4.58	-7.00	-1.30
BDecf (mmol/l)	15.91	7.92	22.52	4.54	0.82	9.75	3.81	0.82	8.45	3.45	0.82	5.61
Apgar 1min	7.67	5	10	8.03	5	10	8.16	5	10	8.45	5	10
Apgar 5min	8.44	7	10	8.81	4	10	8.94	4	10	8.95	4	10
Neonate's weight (g)	3220	2600	4100	3296	2150	3810	3297	2150	3810	3281	2150	3720
Neonate's sex (F/M)		5 / 4			20 / 17			15 / 17			7 / 15	
# cases with pat. outcome	BE: 9; BDecf: 6<br>Apgar at 1min: 0<br>Apgar at 5min: 0			BE: 1; BDecf: 0<br>Apgar at 1min: 0<br>Apgar at 5min: 3			BE: 0; BDecf: 0<br>Apgar at 1min: 0<br>Apgar at 5min: 2			BE: 0; BDecf: 0<br>Apgar at 1min: 0<br>Apgar at 5min: 2		
