# Supplementary material for: Open access intrapartum CTG database
Source: BMC Pregnancy Childbirth. 2014 Jan 13;14:16. doi: 10.1186/1471-2393-14-16 (PMC3898997; doi:10.1186/1471-2393-14-16)
Supplement: Additional file 3 — Table S7 and Table S8. Clinical parameters (risk factors and means of measurement) - vaginal delivery part of the CTG database - pH related. Presentation: O stands for occipital and B for breech. Table S8 Clinical parameters (risk factors and means of measurement) - sectio caesarea delivery part of the CTG database - pH related. Presentation: O stands for occipital and B for breech. [file 1471-2393-14-16-S3.PDF]

Additional files – Table 7: Clinical parameters (risk factors and means of measurement) – vaginal delivery part of the CTG database – pH related. In description of presentation – O stands for occipital and B for breech.

	**pH ≤ 7.05**	**pH > 7.05**	**pH > 7.15**	**pH > 7.25**
**Total number**	**38**	**468**	**412**	**261**
Diabetes	30	364	313	188
Fever	3	29	28	16
Hypertension	0	6	6	4
Preeclampsia	3	39	34	21
Meconium	1	16	14	8
Induced	13	183	165	116
Presentation O	35	425	336	239
Presentation B	1	13	7	7
No progress	7	44	33	27

Additional files – Table 8: Clinical parameters (risk factors and means of measurement) – sectio caesarea delivery part of the CTG database – pH related. In description of presentation – O stands for occipital and B for breech.

	**pH ≤ 7.05**	**pH > 7.05**	**pH > 7.15**	**pH > 7.25**
**Total number**	**9**	**37**	**32**	**22**
Diabetes	9	27	22	12
Fever	1	2	1	1
Hypertension	0	0	0	0
Preeclampsia	0	1	1	1
Meconium	0	2	2	1
Induced	2	23	21	16
Presentation O	9	35	31	21
Presentation B	0	2	1	1
No progress	5	5	4	3
